# Monitoring of Anti-Hepatitis E Virus Antibody Seroconversion in Asymptomatically Infected Blood Donors: Systematic Comparison of Nine Commercial Anti-HEV IgM and IgG Assays

**DOI:** 10.3390/v8080232

**Published:** 2016-08-22

**Authors:** Tanja Vollmer, Juergen Diekmann, Matthias Eberhardt, Cornelius Knabbe, Jens Dreier

**Affiliations:** 1 Herz- und Diabeteszentrum Nordrhein-Westfalen, Universitätsklinik der Ruhr-Universität Bochum, 32545 Bad Oeynhausen, Germany; jdiekmann@hdz-nrw.de (J.Di.); cknabbe@hdz-nrw.de (C.K.); jdreier@hdz-nrw.de (J.Dr.); 2 TMD Gesellschaft für transfusionsmedizinische Dienste mbH, 34117 Kassel, Germany; dr.eberhardt@blutspende-kassel.de

**Keywords:** hepatitis E virus, seroconversion, IgM, IgG, serology, sensitivity

## Abstract

Diagnosis of hepatitis E virus (HEV) is usually determined serologically by detection of the presence of immunoglobulin (Ig)M antibodies or rising anti-HEV IgG titers. However, serological assays have demonstrated a significant variation in their sensitivities and specificities. In this study, we present the systematic comparison of different immunological anti-HEV assays using complete seroconversion panels of 10 virologically confirmed HEV genotype 3 infected individuals. Assay sensitivities were further evaluated by testing serially diluted World Health Organization (WHO) reference reagent for hepatitis E virus antibody and one patient sample infected with HEV genotype 3. Anti-HEV IgM and IgG antibody presence was determined using the immunological assays Wantai HEV IgM/IgG enzyme-linked immunosorbent assay (ELISA) (Sanbio, Uden, The Netherlands), recomWell HEV IgM/IgG (Mikrogen, Neuried, Germany), HEV IgM ELISA 3.0, HEV ELISA, HEV ELISA 4.0, Assure HEV IgM Rapid Test (all MP Biomedicals Europe, Illkirch Cedex, France) and Anti-HEV ELISA (IgM/IgG, Euroimmun, Lübeck, Germany). The assays showed differences regarding their analytical and diagnostic sensitivities, with anti-HEV IgM assays (*n* = 5) being more divergent compared to anti-HEV IgG (*n* = 4) assays in this study. Considerable variations were observed particularly for the detection period of IgM antibodies. This is the first study systematically characterizing serologic assays on the basis of seroconversion panels, providing sample conformity for a conclusive comparison. Future studies should include the assay comparison covering the four different genotypes.

## 1. Introduction

The hepatitis E virus (HEV) is a single-stranded RNA virus, presently grouped into four major genotypes (genotype 1 to 4) with a different geographic distribution, disease pattern and source of transmission: (a) genotype 1 and 2: developing countries, endemic, waterborne; (b) genotype 3 and 4: developed countries, sporadic, zoonotic with hyperendemic genotype 3 areas [1]. HEV infections are increasingly recognized as an emerging disease in developed countries [2,3]. An HEV infection in immunocompetent individuals usually progresses asymptomatically and fewer than 1%–5% of individuals exposed to HEV develop signs of acute hepatitis E [4]. It is certain that HEV has the potential to progress into a fulminant or fatal disease, for example, in individuals with chronic liver disease [5]. Mortality rates range from 0.2% to 4% in the general population [6] reaching 8%–20% in pregnant woman (genotype 1) in developing countries and epidemic settings [7,8]. Chronic infections (genotype 3) have been reported in transplant patients [9,10]. The four HEV genotypes cause very similar antibody responses, suggesting a single serotype [1,11]. Serodiagnosis of hepatitis E demonstrated several limitations in the past, including HEV viremia with a relatively small or without any antibody response in symptomatic and symptom-free individuals [12,13]. The HEV antigens currently used in enzyme immunoassays (EIAs) were produced synthetically or recombinantly in at least two expression systems (*Escherichia coli* and baculovirus) [14], differing in the viral strain origin and the viral gene product (open reading frame (ORF)2 or ORF3) [15]. This resulted in a significant variation in the estimation of seroprevalences, assay sensitivities and specificities [16,17,18,19,20,21,22,23,24]. Therefore, the development of seroconversion and/or genotype-specific panels are of great importance to allow the validation of serological assays [25]. Antigens of most HEV immunoassays were derived from genotype 1 viruses, therefore, their applicability to HEV genotype 3 infections is indeterminate [17]. Only a handful of studies analyzed the performance of serological assays with different genotypes [19,22,23]. Furthermore, previous studies assessed the diagnostic sensitivity of either HEV-specific immunoglobulin (Ig)G or IgM solely with a cohort consisting of a single sample for each patient [16,22,24], whereas studies also including the analytical sensitivity are rare [22,26]. We recently described the natural course of asymptomatic genotype 3 infection [27], demonstrating to a very limited extent, that the diagnostic window depends on the serological assay used. Consequently, the present study focuses on the systematic comparison of the diagnostic sensitivity of different commercially available anti-HEV serological assays by using unique samples of 10 seroconversion panels of virologically confirmed HEV genotype 3 infected individuals. Furthermore, the analytical sensitivity was compared by testing serially diluted World Health Organization (WHO) reference reagent for hepatitis E virus antibody and a plasma sample of one virologically confirmed HEV genotype 3 infected individual. 

## 2. Material and Methods

### 2.1. Specimen

A total of 16,125 individual donors were routinely screened for the presence of HEV RNA by the Uni.Blutspendedienst OWL (Herz- und Diabeteszentrum Nordrhein-Westfalen, Bad Oeynhausen, Germany), recovering 13 HEV RNA positive donors, between July and September 2011 [28]. Retrospectively, residual plasma samples of previous and follow-up donations spanning the initial HEV RNA positive donation were collected and available in continuous intervals for 10 blood donors (all male). The number of specimens varied from eight to 23 samples for each panel. Samples covered a time period from 0 to 42 days maximum before seroconversion, a minimum distance between time points of three days and a maximum distance of 42 days (mean: 10 ± 9 days). All donors underwent a pre-donation medical examination without conspicuousness and negated current diseases or any known risk factors for viral infection. Detection of HEV in plasma samples was performed using the RealStar HEV RT-PCR kit (Altona Diagnostic Technologies, Hamburg, Germany), as described previously [28]. The study protocol conformed to the ethical guidelines and was approved by the ethics committees of the institution. Informed consent was obtained from each donor.

### 2.2. Serological Testing

Nine commercially available immunoassays (five anti-HEV IgM assays, four anti-HEV IgG assays) and one anti-HEV all-AB assay from four manufacturers were compared in this study based on their application in previous studies [29] and common use in German laboratories. The specifications of the immunoassays used for comparison are described in Table 1, enzyme immunoassays were performed according to the manufacturer’s instructions. Consideration of the European literature dealing with HEV IgG seroprevalence indicates that Wantai (Sanbio, Uden, The Netherlands), Mikrogen (Mikrogen GmbH, Neuried, Germany) and MP diagnostic (MP diagnostic Europe, Illkirch Cedex, France) assays were the most frequently used assays. 

Twofold dilution series of the WHO Reference Reagent for hepatitis E virus antibody (WHO reference reagent for hepatitis E virus antibody (WHO-Ref) Code: 95/584, NIBSC, Hertfordshire, UK, dilution 1:1 to 1:256, range: 0.4 to 100 WHO units/mL, human serum NIBSC code: 95/584) and the HEV IgM and IgG positive sample of donor 6 (day 55, dilution 1:1 to 1:128) were prepared with human plasma negative for anti-HEV IgM, anti-HEV IgG, anti-HBs, HBsAg, anti-HCV and anti-HIV1/2 to compare the sensitivity and linearity of all EIAs. The concentration of anti-HEV IgG in the donors’ samples was calculated by quadruplicate analysis using the Anti-HEV ELISA (IgG KitEU, Euroimmun). Quantification of this assay is based on the WHO-Ref and results were provided by the manufacturer in IU/mL. The corresponding concentrations for the different dilution steps were as follows: 16.8 IU/mL (1:1), 8.4 IU/mL (1:2), 4.2 IU/mL (1:8), 2.1 IU/mL (1:8), 1.1 IU/mL (1:16), 0.5 IU/mL (1:32), 0.3 IU/mL (1:64) and 0.1 IU/mL (1:128). Although the WHO-Ref was developed for the quantification of total IgG anti-HEV, the standard also contains small amounts of IgM anti-HEV [15,31]. Samples were analyzed in triplicate. Continuous plasma samples were analyzed in triplicate for the presence of HEV-specific antibodies with all immunoassays. The duration of seropositivity was displayed as half of the interval between last negative and first positive sample because the seropositivity starts before the first positive sample and lasts beyond the last positive sample. To compare the diagnostic sensitivity of all assays, the day of the first detection of anti-HEV antibodies, the different IgM or IgG assays were ranked as follows: first positive detection: score 3; second positive detection: score 2; third positive detection: score 1; no positive detection: score 0.

### 2.3. Statistical Analysis

Calculation of all values and statistical analysis was performed using the GraphPad Prism 5.0 software (GraphPad Software, San Diego, CA, USA). Linearity of assays was determined by linear regression analyses. We used Spearman correlation coefficients to assess correlations between variables. *p*-values < 0.05 were considered significant. 

## 3. Results

### 3.1. Comparison of the Analytical Sensitivities by Analysis of Serial Dilution Samples

A twofold serial dilution series of the WHO-Ref and one HEV IgM and IgG positive donor sample were analyzed in parallel in order to evaluate the analytical sensitivity of the different anti-HEV ELISAs (Table 2, also see Figures S1 and S2). The WHO Standard revealed differences in analytical sensitivity for the four different anti-HEV IgM tests, showing the positive detection of IgM antibodies until dilution factor 1:8 using the MP-Bio assay, compared to 1:4 for the other assays (Wantai, Mikrogen, Euroimmun). The differences in the analytical sensitivity of the diluted donor sample range from 1:64 (MP-Bio), 1:32 (Mikrogen) to 1:16 (Wantai, Euroimmun). This represents a 2-dilution step (WHO-Ref) and a 1- or 2-dilution step (donor sample) difference between the most sensitive assay (MP-Bio) and the other assays. 

The differences in the analytical sensitivities of the three different anti-HEV IgG assays were lower. The MP-Bio and Euroimmun assays showed an analytical sensitivity in the range of 1.5 IU/mL (dilution 1:64) for the WHO-Ref or 1.1 IU/mL (dilution 1:16) for the diluted donor sample. The Mikrogen assay revealed a marginally lower sensitivity of 3.1 IU/mL (dilution 1:32) for the WHO-Ref and a comparable sensitivity of 1.1 IU/mL for the diluted donor sample. Remarkably, the Wantai assay still indicated the presence of anti-HEV IgG antibodies corresponding to a titer of 0.4 IU/mL (WHO-Ref) and 0.6 IU/mL (diluted donor sample). In summary, the differences observed regarding the analytical sensitivities of the anti-HEV IgG assays include, at most, one dilution step and all assays provide sensitivities within the range of 1.1–3.1 IU/mL, with the exception of the Wantai assay. No considerable differences regarding the sensitivity were observed between a genotype 1 (WHO-Ref) or genotype 3 (donor sample) infected individual. Finally, consistent linearities were observed for both dilution series with all assays applied (Table 2).

The analytical sensitivity of the All antibody HEV ELISA 4.0 (MP Biomedical, Figure S1) agreed with the results obtained for the MP Bio IgG-specific assay (dilution factor 1:64) for the WHO-Ref; the sensitivity of 1:32 observed for the diluted donor sample is provided between the sensitivity of the MP BioIgM specific assay (1:64) and the IgG specific assay (1:16).

The Euroimmun anti-HEV IgG assay is the only assay providing antibody titers quantified based on the WHO-Ref and comparison of quantitative results obtained for the WHO-Ref showed a good correlation (Spearman rank correlation coefficient r = 1.00, *p* < 0.0001) between the provided and measured IgG concentration. 

### 3.2. Comparison of the HEV Seroconversion Panels

**Figure 1:**
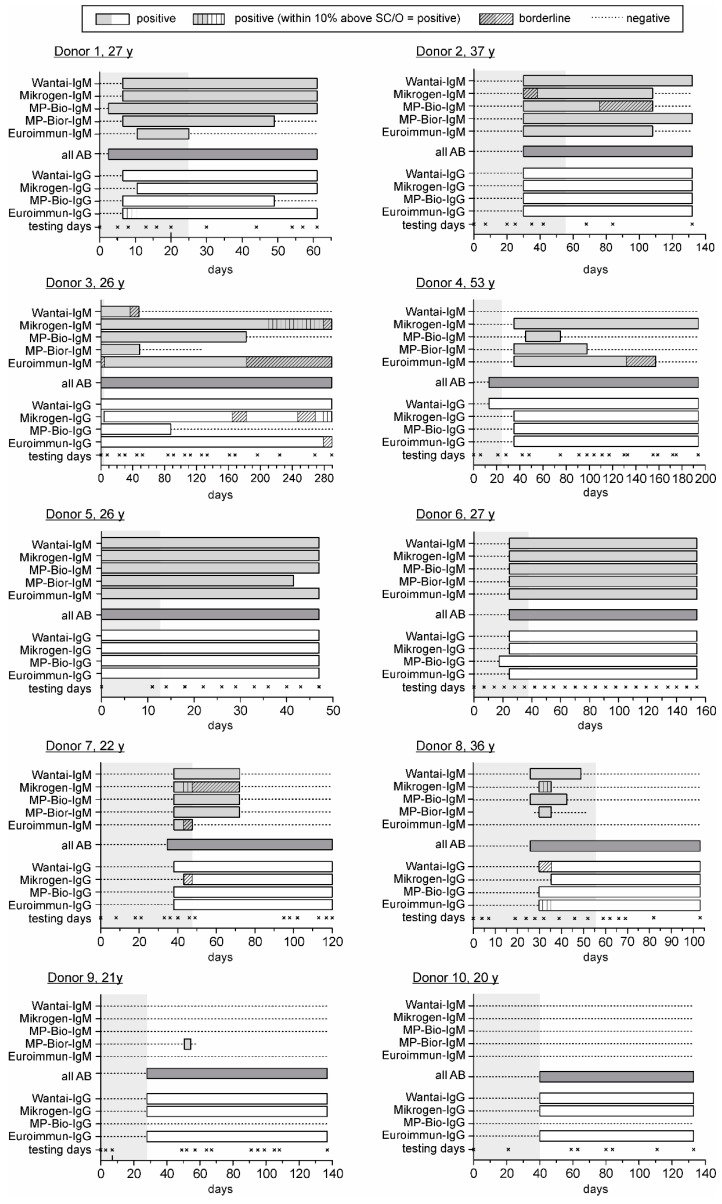
Comparison of different anti-hepatitis E virus (HEV) assays regarding the course of immune response during seroconversion of 10 blood donors with acute HEV infection. The course of immune response of 10 blood donors with autochthonous HEV infection is displayed, determined by 10 different commercially available anti-HEV immunoassays. The day of the detection of HEV RNA by PCR screening was defined as day 0 [28], confirmation of the presence of HEV RNA is indicated by gray shading. The period of positive testing results is displayed by light grey bars for the five HEV immunoglogulin (Ig)M-specific assays, by white bars for the four HEV IgG-specific assays and by dark grey bars for the HEV all antibody assay (see Table 1 for the encoding of the kits). Bars are starting at half of the interval between the last negative and first positive sample and last until half of the interval between last positive and first negative sample. The Assure HEV IgM Rapid Test (MP-Bior-IgM) was only performed with limited samples for donor 3 (day 0–126), donor 5 (day 0–40), donor 8 (day 28–52) and donor 9 (day 0–57). SC/O: signal-to-cutoff; AB: antibody.

Clinical follow-up samples of 10 HEV RNA positive blood donors were collected over different time periods of 47 to 280 days to compare the diagnostic sensitivity of the different serological tests. The results of all assays are displayed in Figure 1, reflecting the course of anti-HEV-specific antibodies differently. Remarkably, the Wantai HEV IgM assay was not able to confirm IgM seropositivity of samples of donor 4, whereas all other assays notify a clear IgM seropositivity.

On the one hand, all IgM or IgG assays were ranked in relation to their supply of the first distinct positive result after the initial detection of viremia among themselves to compare the diagnostic sensitivity (Table 3). The differences observed regarding the detection of anti-HEV IgM antibodies in seroconversion panels of donors 1–8 only span the previous or subsequent testing time points correlating with maximum differences regarding the first anti-HEV IgM detection ranging from four to eight days. Noticeably, only the MP-Bio rapid assay detected anti-HEV IgM antibodies in one sample of donor 9. Indeed, for donors 9 and 10, samples were not available within the timeframe where early IgM seroconversion most likely occurred. Subsequently, the evaluation of assays using the score system introduced indicates that the MP-Bio rapid assay demonstrated the highest sensitivity (score 25), followed by the MP-Bio and Mikrogen assays (score 23 and 21) and the Wantai and Euroimmun assays (score 20 and 18, Table 3). 

No differences in the detection of anti-HEV IgG were observed for the seroconversion panels of donors 2 and 5. The seroconversion panels of donors 1, 3, 6–10 cover at least differences only between the previous or subsequent testing time point correlating with maximum differences regarding the first anti-HEV IgG detection ranging from three to 21 days. The most noticeable differences could be observed with samples of donor 4, where the Wantai assay detected IgG antibodies considerable earlier compared to the other assays applied, although IgM antibodies were also not detectable with all assays applied. Only the HEV ELISA 4.0 (all antibodies) revealed a positive detection of anti-HEV antibodies. Interestingly, the MP-Bio assay was not able to detect IgG antibodies in any sample from donors 9 or 10, or from a certain time point in donors 1 and 3, although the HEV ELISA 4.0 (all antibodies) provided by the same company detected HEV antibodies concordant with the other IgG-specific assays. The evaluation of assays using the score system introduced disclose that the Wantai and Euroimmun assays demonstrated the highest sensitivity (score 28), followed by the two other assays with almost equal scores (Table 3). 

On the other hand, the evaluation of the detection period of anti-HEV IgM showed noticeable, donor-depending variations among the IgM assays (donors 1–4, 7 and 8). For example, obvious differences were seen using the Wantai assay, in particular for the seroconversion panels of donor 8, where this assay determined the presence of anti-HEV IgM antibodies for a considerable longer time period than any other assay. In contrast, the Mikrogen, MP-Bio and Euroimmun assays determined a significantly longer IgM positivity compared to the Wantai assay in samples of donor 3. Additionally, this assay was not able to detect anti-HEV antibodies in samples of donor 4 at any time, whereas the Mikrogen assay showed IgM positivity for more than 150 days. Moreover, the signal levels of assays determining the presence of anti-HEV IgM differ; for example, in samples of donor 2, the Wantai, MP-Bio-rapid and Euroimmun assays provide clear positive results, whereas the Mikrogen and MP-Bio assaysassay determine borderline results. 

Consideration of seroconversion panels from donors 1 and 3 suggests that IgG antibodies seem to disappear using the MP-Bio (donor 1) or the Mikrogen, MP-Bio or Euroimmun assays (donor 3).

## 4. Discussion

Currently there are various commercially available and in-house serological anti-HEV diagnostic tests. Unfortunately, a widespread variability of assay sensitivities and specificities has complicated the collection of reliable and comparable data. The comparison of the IgG seroprevalence, for example, among blood donors in the same region in France revealed significantly different rates using Wantai and the MP Bio IgG assays (52.5% vs. 16.6%, [20,32]). 

Avellon and colleagues recently chose a new strategy to determine the sensitivity by including specific IgM and IgG sample panels with high and low antibody concentrations exceeding the traditional dilution of single samples [26]. Complementarily, we investigated the diagnostic sensitivity by using unique samples of 10 seroconversion panels of virologically confirmed HEV genotype 3 infected individuals. For the first time, our study systematically evaluates the analytical and diagnostic sensitivity in parallel, revealing deviating performances of the analytical and diagnostic sensitivities for the particular assays. 

To achieve a more simple and better classification and comparison of the obtained results in this study within the context of the known literature, the results of analytical and diagnostic sensitivities obtained in this study and former comparative studies were summarized in Table 4 (IgM assays) and Table 5 (IgG assays). Only assays used in the current study were considered. The closer observation of the different studies revealed that very heterogeneous sample cohorts were used. This aspect makes an objective comparison difficult, especially for the diagnostic sensitivity. The determined values for sensitivity and specificity are valid only for the sample cohort of the respective study and a statement regarding the true positive and true negative rates remains open. 

In our study, the evaluation of the analytical sensitivities of IgM assays disclose that the MP-Bio assay has the highest sensitivity (WHO Ref.: MP-Bio > Wantai/Mikrogen/Euroimmun), donor sample: MP-Bio > Mikrogen > Wantai/Euroimmun. By contrast, the highest diagnostic sensitivity in the head-to-head comparison of seroconversion panels was obtained with the. MP-Bio rapid assay, followed by MP-Bio > Mikrogen > Wantai > Euroimmun. The comparison of the analytical sensitivities of other studies disclosed inverse orders (genotype 1: MP-Bio/Wantai > Mikrogen [22]; genotype 3: Wantai > Mikrogen > MP-Bio [22] vs. Mikrogen > Wantai > Euroimmun > MP-Bio [26], Table 4). Surprisingly, the immunographic IgM assay was more sensitive than the microplate assays compared in this study. This result could mostly be explained by the exclusive detection of IgM antibodies in samples of donor 9 by this assay. An important factor and also a potential shortcoming of this test is the result interpretation, which is sometimes difficult due to very faint bands and a strong dependence on the performing experimenter. The expectancy of the experimenter in routine clinical use might influence the result interpretation, followed by the chance of missing or overinterpreting an IgM positive sample due to very faint bands. However, seen from the angle of major outbreaks in developing nations, a simple and non-laborious test enabling early detection of IgM antibodies would enhance the management of HEV infection [37]. 

Additionally, the inter-donor variability regarding the persistence of IgM is an interesting observation. In some cases antibodies showed persistence with a duration greater than 100 days (D3, D4, D6) whereas in other cases antibodies continuously decreased and disappeared. We recently investigated the natural course of asymptomatic HEV infection, but data from a large cohort of asymptomatically HEV infected individuals are lacking. To date we have no reliable explanation for this observation. Indeed, differences regarding the diagnostic sensitivities are one potential explanation partly covering these findings. Secondarily, a reduced assay specificity in samples of individuals after they had experienced HEV infection could not be excluded and the clinical implication of these differences needs to be evaluated systematically in future approaches. 

Variabilities of the analytical and diagnostic sensitivities were also noticed for the IgG assays (Table 4), but in this study at a lesser extent than for IgM assays. The highest analytical and diagnostic sensitivity was observed for the Wantai IgG assay. The order of analytical sensitivities for the WHO-Ref samples was Wantai > MP-Bio/Euroimmun > Mikrogen compared to Wantai > Mikrogen/MP-Bio/Euroimmun for the diluted donors sample. Consecutively, the order of the diagnostic sensitivity is opposite to the analytical sensitivities for the WHO-Ref samples Wantai/Euroimmun > Mikrogen > MP-Bio, probably due to the missing detection of anti-HEV IgG antibodies by the MP-Bio assay in subsequent samples of donors 1, 3, 9 and 10, which is discussed separately below. Besides other assays not included in this study, Pas et al. [22] systematically compared the analytical sensitivity for the WHO-Ref of the Mikrogen assay (3.16 IU/mL), MP-Bio assay (2.63 IU/mL) and the Wantai assay (0.69 IU/mL), and the results were fairly comparable to the results determined in this study (Table 2 and Table 4). The study by Bendall et al. [17] determined comparable sensitivities of the MP Bio and Wantai IgG assays (2.5 vs. 0.25 WHO units). Interestingly, the study by Norder et al. [35] showed a higher sensitivity of the Mikrogen assay (0.9 IU/mL).

Furthermore, they observed a longer positive postinfection period for the Wantai assay and a higher diagnostic sensitivity (98% vs. 53%). Nonetheless, inverse orders were again noticed for the analytical sensitivities obtained by genotype 3 samples (Table 4) as follows: (a) this study: Wantai > Mikrogen/MP-Bio/Euroimmun vs. (b) Mikrogen > Wantai > MP-Bio [22] vs. (c) Wantai/Mikrogen > MP-Bio > Euroimmun [26]. One explanation for the lack of concordance observed in the different studies might by the fact that different kit versions were tested (improvements by the manufacturers). For example, the Mikrogen company released a new version of their IgM assay in 2012, which has shown a higher sensitivity than the old version (52% vs. 74%, [22]). We also applied both tests on the WHO-Ref samples and all seroconversion panels, confirming these results (data not shown). Moreover, the assay cut-off value determined by the manufacturer strongly influence the sensitivity of the assays and, in consequence, the seroprevalence rates. Recently, the cut-off value of the Euroimmun IgG-assay was adjusted from 2.0 IU/mL to 1.0 IU/mL, based on the results of several comparative studies on seroprevalence rates using different assays. This impact is also detectable in this study since the sensitivity determined using the dilution series increased by one dilution step (e.g., WHO sample: 3.1 IU/mL (old cut-off) to 1.6 IU/mL (new cut-off). Likewise, seroconversion was detected at least one sampling time point earlier in four donors (donor 1: day 13 to day 8, donor 3: day 8 to day 0, donor 8: day 39 to 32 and donor 9: day 52 to 49, data not shown). Furthermore, the usage of different samples or sample panels as mentioned above had a great impact on the result outcome. For instance, a considerably lower IgG sensitivity of <50% has been observed in some studies [16,23], probably caused by the testing of HEV RNA-positive samples prior to seroconversion. This aspect further enforces the demand for defined sample material with known serostatus in order to obtain a high reproducibility and informative value and the collection of comparable and reliable data. Accordingly, the comparative testing on the basis of a panel of serum samples to evaluate the accuracy of different assays was demanded by several authors [25,38,39]. 

In compliance with these demands, the application of the WHO-Ref supported the assessment of assay sensitivities, but does not fully solve this issue [26]. The application of panels of follow-up samples previously used in two studies [17,26] further advance the termination of this problem, but defined panels were currently not available. The unique systematic comparison of serological assays using seroconversion panels presented in this study provides further reliable insights into the comparability of different serological assays. These seroconversion panels were immediately available, either for the validation of serological HEV assays or for the validation of PCR assays [40]. The original serum used for WHO-Ref was collected from a US patient after they had travelled to India (most likely infected with HEV genotype 1 [14]), whereas the plasma used for the dilution studies was obtained from donor 6, who was infected with genotype 3 [28]. The consistent linearity observed for both dilution series with all assays introduced supports the single serotype character of the four different HEV genotypes [1,11,41]. In conclusion, we did not observe a reduced sensitivity for the detection of antibodies in genotype 3 infected individuals with assays coated with genotype 1, 2 and/or 4 antigens, consistent with other studies [19,22,23]. 

Unfortunately, the sensitivity of the assays in asymptomatic patients may not reflect the sensitivity in symptomatic patients, since the clinical symptoms seem to be linked to the immune response during HEV infection. In line with this, chronic infections are frequently asymptomatic in immunocompromised patients and are associated with a moderate increase in alanine aminotransferase. We certainly observed some conspicuous characteristics of the Wantai IgM and the MP-Bio IgG assay in our seroconversion panels. Unusually, the Wantai assay was not capable of detecting IgM antibodies (donor 4) whereas the MP-Bio assay was not capable of detecting IgG antibodies at all (donor 9/10) or from a certain time point (donors 1 and 3). A potential prozone effect was excluded by the analysis of 1:2 and 1:4 diluted aliquots from the samples involved (data not shown). Indeed, the all antibody ELISA distributed by the same company than the IgG-MP assay provided positive results despite the absence of IgM antibodies. The comparison of results obtained for the final concentration of IgG antibodies for the last testing day using the Euroimmun assay revealed IgG titers of 4.41 IU/mL (donor 9) and 5.84 IU/mL (donor 10); nevertheless, the lowest IgG titer with positive results for all assays amounted to 5.4 IU/mL (donor 7). Therefore, we could not observe a correlation between the final IgG concentration and the disappearance of IgG antibodies. A poorer sensitivity in general contradicts the results obtained with the serial dilutions of the WHO-Ref and samples of donor 6. Previous studies have shown that the duration of anti-HEV IgG can vary individually from six months to 14 years, but disappearance of HEV IgG antibodies has also been observed [12,41,42,43]. Consideration of the results of different IgG assays for samples of donor 3 revealed concordant alternating positive and negative IgG results after 120 days of observation, suggesting a potential decrease of anti-HEV IgG antibodies, at least close to or below the detection limits of the respective assays. 

The clinical relevance of the assay sensitivities required is indeterminate to date, but the assay specificity also plays an important role. Consideration of the European literature showed that the anti-HEV Wantai assay revealed higher seroprevalence rates compared to all the other assays and has been regarded by many as the “gold standard” in the field [44]. Perhaps one reason for this is the validated sensitivity for detecting infection in proven cases of 98% [17]. However, some observers contended that the high seroprevalence results produced by the Wantai assay stretch biological plausibility and are simply a reflection of the assay’s poor specificity. In accordance with all other commercial assays, the Wantai assay has not been fully assessed in terms of specificity due to the absence of adequate and comparable examination material. Assay specificities vary strongly depending on the assay used and the specificity panel determined: (a) MP Bio (IgM): 93%, Mikrogen (IgM): 95.6% [18]; (b) MP Bio (IgM) 74%, MP Bio (IgG) 86% [21]; (c) Wantai (IgM): 100%, Wantai (IgG): 97.8% [16]; (d) MP Bio (IgM): 99.5% and MP Bio rapid (IgM): 100% [19]. Specificity panels most often consisted of samples from healthy blood donors [16,22], but also samples from individuals, for example, with a positive rheumatoid factor, positive anti-HAV IgM or other acute infections were used [22,33]. Indeed, the usage of blood donor samples limits the informative value of specificity and also sensitivity panels, since 0.08% of blood donors undergo an acute asymptomatic infection [28], falsifying the results of anti-HEV assays depending on their status of infection. Nevertheless, the application of different assays on the same samples of an in-house specificity panel as presented by Pas and colleagues [22] allows the comparison of specificities for the different IgM assays (84%: MP Biomedicals, 98%: Wantai and 99%: Mikrogen). Nonetheless, the selection of samples for specificity panels is currently an individual decision since no defined specificity panel is presently available. The question regarding true negative or positive samples in these panels remains open. Therefore, we refrain from the establishment of an in-house specificity panel. The design of our study does not allow the calculation of the specificity of the different assays, and this aspect was also not intended in the study. An international collaborative effort is currently underway to establish a defined panel for the assessment of assay specificities to overcome this limitation [25]. 

In conclusion, we have shown that different anti-HEV assays differ regarding their sensitivities for the detection of HEV genotype 3 infection, demonstrating that, in this study, anti-HEV IgM assays are more divergent than anti-HEV IgG assays. Differences observed between the analytical and diagnostic assay sensitivities enforce the analysis of seroconversion panels in addition to end-point titration studies for an effective assay evaluation. 

## Figures and Tables

**Table 1:** Overview of specifications for the different HEV immunoassays.

Anti-HEV ELISA	Assay Type	Antigen/Origin	Analysis and Serostatus Interpretation
***IgM assays***			
Wantai HEV IgM ELISA (Sanbio ^1^)	qualitative, µ-chain capture	recombinant antigen ORF-2 C-terminal, genotype 4 [30]	- negative: C.O. < 0.9 - borderline: C.O. 0.9–1.1 - positive: C.O. ≥ 1.1
recomWell HEV IgM (Mikrogen GmbH ^2^), new version (08/2012)	quantitative, indirect	recombinant antigen ORF-3 C-terminal, genotype 1, 2 and 3 [22]	- negative: <20 U/mL - borderline: ≤20 to ≤24 U/mL - positive: >24 U/mL
HEV IgM ELISA 3.0 (MP Biomedicals ^3^)	qualitative, indirect	1 recombinant antigen, ORF-2 C-terminal (Chinese strain), genotype 1	- negative: C.O. < 0.4 + NRC - positive: C.O. ≥ 0.4 + NRC
Assure HEV IgM Rapid Test (MP Biomedical ^3^)	qualitative, reverse-flow immunochr	1 recombinant antigen, ORF-2 C-terminal (Chinese strain), genotype 1	- positive: test-line and control-line - negative: control-line
Anti-HEV ELISA (IgM, Euroimmun ^4^)	qualitative, indirect	1 recombinant antigen ORF-2 (USA strain), genotype 3	- ratio: extinction sample/calibrator - negative: ratio < 0.8 - borderline: ratio ≥ 0.8 to ≤ 1.1 - positive: ratio ≥ 1.1
***All antibody assays (IgA, IgM, IgG)***			
HEV ELISA 4.0 (MP Biomedicals)	qualitative, direct	1 recombinant antigen, ORF-2 C-terminal (Chinese strain), genotype 1	- negative: C.O. < 0.4 + NRC - positive: C.O. ≥ 0.4 + NRC
***IgG assays***			
Wantai HEV IgG ELISA (Sanbio ^1^)	qualitative, indirect	recombinant antigen ORF-2 C-terminal, genotype 4 [30]	- negative: C.O. < 0.9 - borderline: C.O. < 0.9–1.1 - positive: C.O. ≥ 1.1
recomWell HEV IgG (Mikrogen GmbH ^2^)	quantitative, indirect	recombinant antigen ORF-2 C-terminal, genotype 1 and 3	- negative: <20 U/mL - borderline: ≤20 to ≤24 U/mL - positive: >24 U/mL
HEV ELISA (MP Biomedicals ^3^)	qualitative	3 recombinant antigens, ORF 2 and ORF 3 (Burmese, Mexican strains), genotype 1 and US type 2	- negative: C.O. < 0.5 + NRC - positive: C.O. ≥ 0.5 + NRC
Anti-HEV ELISA (IgG, Euroimmun ^4^)	quantitative (IU/mL), indirect	1 recombinant antigen ORF-2 (USA strain), genotype 3	- negative: <0.8 IU/mL - borderline: ≥0.8 to <1.1 IU/mL - positive: ≥1.1 IU/mL

^1^ Sanbio B.V., Uden, The Netherlands; ^2^ Mikrogen GmbH, Neuried, Germany; ^3^ MP Biomedicals Europe, Illkirch Cedex, France (previously distributed by Genelabs); ^4^ Euroimmun AG, Lübeck, Germany. HEV: hepatitis E virus; ELISA: enzyme-linked immunosorbent assay; Ig: immunoglobulin; ORF: open reading frame; IU: international unit; C.O.: cut-off; NRC: non-reactive control.

**Table 2:** Comparison of the detection limits and linearity of the different anti-HEV antibody assays.

Assay	WHO-Ref (GT 1) IU/mL (Dilution)		Donor Sample (GT 3) (Dilution)	
	Detection Limit	Linearity (R^2^) *	Detection Limit	Linearity (R^2^) *
***Anti-HEV IgM***				
Wantai	1:4	0.9937	1:16	0.9994
Mikrogen	1:4	0.9474	1:32	0.9199
MP-Bio	1:8	0.9950	1:64	0.9824
Euroimmun	1:4	0.9499	1:16	0.9186
***All-AB***				
MP-Bio	1:64	0.9969	1:32	0.9819
***Anti-HEV IgG***				
Wantai	0.4 (1:256)	0.9324	0.6 (1:32)	0.9857
Mikrogen	3.1 (1:32)	0.9197	1.1 (1:16)	0.9799
MP Biomedical	1.5 (1:64)	0.9508	1.1 (1:16)	0.9490
Euroimmun	1.5 (1:64)	0.9934	1.1 (1:16)	0.9973

* Linear regression analysis was performed using the GraphPad Prism 5.0 software.

**Table 3:** Comparison of the day determining the first detection of IgM and IgG antibodies (exclusively positive results) by the different serological assays.

		IgM													IgG									
Donor		Wantai		Mikrogen		MP-Bio		MP-Bio Rapid		Euroimmun		Range Detection	Maximum Difference		Wantai		Mikrogen		MP-Bio		Euroimmun		Range Detection	Maximum Difference
		Day	SC	Day	SC	Day	SC	Day	SC	Day	SC				Day	SC	Day	SC	Day	SC	Day	SC		
1		8	2	8	2	5	3	8	2	13	1	5–13	8		8	3	13	2	8	3	8	3	8–13	5
2		35	3	42	2	35	3	35	3	35	3	35–42	7		35	3	35	3	35	3	35	3	35	-
3		0	3	0	3	0	3	0	3	8	2	0–8	8		0	3	8	2	0	3	0	3	0–8	8
4		-	0	42	3	48	2	42	3	42	3	42–48	6		21	3	42	2	42	2	42	2	21–42	21
5		0	3	0	3	0	3	0	3	0	3	0	-		0	3	0	3	0	3	0	3	0	-
6		28	3	28	3	28	3	28	3	28	3	28	0		28	2	28	2	21	3	28	2	21–28	7
7		40	3	40	3	40	3	40	3	40	3	40	0		40	3	49	2	40	3	40	3	40–49	9
8		28	3	32	2	28	3	32	2	-	0	28–32	4		39	2	39	2	32	3	32	3	32–39	7
9		-	0	-	0	-	0	52	3	-	0	52	-		49	3	49	3	-	0	49	3	49	0
10		-	0	-	0	-	0	-	0	-	0	-	-		59	3	59	3	-	0	59	3	59	-
SC *		20		21		23		25		18		-	-		28		24		23		28		-	-

* SC: score, the different IgM or IgG assays were ranked as follows: first positive detection: score 3; second positive detection: score 2; third positive detection: score 1; no positive detection: score 0.

**Table 4:** Anti-HEV IgM sensitivities (analytical sensitivity, diagnostic sensitivity) and specificities of assays compared in this study with previous comparative reports.

Study Cohort Sens./*Spec.* (No. Patients/Total)	Assay	Analytical Sensitivity		Sens. (%)	Spec. (%)	Ref.
		GT 1	GT 3			
blood donors, GT3 (10/145) WHO Ref., GT1	Wantai	dilution 1:4	dilution 1:16	(+)	n.d.	this study
	Mikrogen	dilution 1:4	dilution 1:32	+	n.d.	
	MP-Bio	dilution 1:8	dilution 1:64	++	n.d.	
	Euroimmun	dilution 1:4	dilution 1:16	(-)	n.d.	
	MP-Bio rapid	n.d.	n.d.	+++	n.d.	
patients: confirmed HEV infection, GT1 + 3 (36/88) *cohort a (98/98)*	Wantai	titer >64.000	titer >64.000	75	>99	[22] ^a^
	Mikrogen	titer 32.000	titer 16.000	74	99	
	MP-Bio	titer >64.000	titer 4.000	74	84	
patients: acute hepatitis, GT3 (14/52)	Wantai	n.d.	n.d.	65.4	n.d.	[26] ^a^
	Mikrogen			75.0	n.d.	
	MP-Bio			59.6	n.d.	
	Euroimmun			61.5	n.d.	
patients: acute hepatitis, GT1 + 3 (50/50) *patients: HEV-RNA/IgG negative (406/406)*	MP Bio	n.d.	n.d.	88	99.5	[19]
	MP Bio rapid			82	100	
patients: a: suspected (71/71) b: suspected RNA+ (35/35) c: confirmed HEV (55/55) *patients: rheumatic factor+, anti-HAV + (104/104)*	Wantai	n.d.	n.d.	a: 83.08 b: 97.14 c: 87.27	100	[33]
	MP-Bio			a: 78.46 b: 74.29 c: 67.27	89.11	
patients: HEV infection RNA+, GT1-4 (50/50) *cohort b (229)*	Mikrogen	n.d.	n.d.	92	95.6	[18]
	MP-Bio			72	93	
patients: suspected HEV infection, GT 1, 3, 4 (309/309) *same cohort (309/309)*	Mikrogen	n.d.	n.d.	93.3	88.4	[34]
	MP-Bio			80.0	86.1	
patients: a: immunocompromised (40/40) b: immunocompetent (44/44) *blood donors, HEV-RNA/IgG negative (223/223)*	Wantai	n.d.	n.d.	a: 85 b: 97.7	99.6	[16] ^a^
patients: a: symptomatic (82/82) b: asymptomatic (174/174) *cohort c (496/496)*	MP-Bio	n.d.	n.d.	a: 42 b: 72	74	[21] ^a^
patients: suspected HEV infection, GT unknown (66/66), WHO Ref., GT1, dilution two patient samples * *same cohort (66/66)*	Mikrogen	16 IU/mL	*1/53, *1/43	38	99	[35] ^a^
	Euroimmun	24 IU/mL	*1/35, *1/22	24	100	
patients: a: immunocompromised (30/30) b: immunocompetent (30/30) *cohort d (60/60)*	Wantai	n.d.	n.d.	a: 83.3 b: 96.7	96.7	[36]

^a^ further comparison to other assays not mentioned in this study; HEV genotyping was performed by sequencing analysis described in the individual studies; cohort a: acute EBV/CMV/PVB19/HAV/HBV/HCV infection, blood donors, transplant recipients; cohort b: blood donors, household contacts of HEV infected patients, acute HAV/HBV/HCV infection; cohort c: individuals from an unaffected village, no acute illness; cohort d: HEV-RNA−, acute EBV/CMV infection. Sens.: sensitivity, Spec.: specificity, n.d. not determined. Rating: +++: most sensitive assay, ++: second most sensitive assay, +: third most sensitive assay, (+): fourth most sensitive assay, (-): fifth most sensitive assay.

**Table 5:** Anti-HEV IgG sensitivities (analytical sensitivity, diagnostic sensitivity) and specificities of assays compared in this study with previous comparative reports.

Study Cohort Sens./*Spec.* (No. Patients/Total)	Assay	Analytical Sensitivity		Sens. (%)	Spec. (%)	Ref.
		GT 1	GT 3			
blood donors, GT3 (10/145) WHO Ref., GT1	Wantai	0.4 IU/mL	0.6 IU/mL	+++	n.d.	this study
	Mikrogen	3.1 IU/mL	1.1 IU/mL	++	n.d.	
	MP-Bio	1.5 IU/mL	1.1 IU/mL	+	n.d.	
	Euroimmun	1.5 IU/mL	1.1 IU/mL	+++	n.d.	
patients: confirmed HEV infection, GT1 + 3 * WHO Ref., GT1	Wantai	0.69 IU/mL titer >12,800 *	titer 1.600 *	n.d.	n.d.	[22] ^a^
	Mikrogen	3.16 IU/mL titer >12,800 *	titer 3.200 *		n.d.	
	MP-Bio	2.63 IU/mL titer 3200 *	titer 100 *		n.d.	
patients: seroconversion after acute hepatitis (GT3) (10/40)	Wantai	n.d.	n.d.	72.5	n.d.	[26] ^a^
	Mikrogen			72.5	n.d.	
	MP-Bio			70.0	n.d.	
	Euroimmun			57.5	n.d.	
patients: acute hepatitis (15/15)	Wantai	n.d.	n.d.	93	n.d.	[23] ^a^
	MP Bio	n.d.	n.d.	53	n.d.	
patients: a: acute hepatitis b: follow-up acute hepatitis (18/50)	Wantai	0.25 IU/mL	n.d.	a: 98 b: 100	n.d.	[17]
	MP-Bio	2.5 IU/mL		a: 53 b: 50	n.d.	
patients: suspected HEV infection (309) *same cohort (309/309)*	Mikrogen	n.d.	n.d.	86.7	77.9	[34]
	MP-Bio			73.3	65.3	
patients: a: immunocompromised (40/40) b: immunocompetent (44/44) *blood donors, HEV-RNA/IgG negative (223/223)*	Wantai	n.d.	n.d.	a: 45 b: 93.2	97.8	[16] ^a^
patients: a: symptomatic (82/82) b: asymptomatic (174/174) *cohort c (496/496)*	MP-Bio	n.d.	n.d.	a: 51 b: 89	86	[21] ^a^
blood donors patients: suspected hepatitis E, liver disease, liver transplantation, GT unknown (216/216) WHO Ref., GT1	Mikrogen	0.9 IU/mL	n.d.	62	99	[35] ^a^
	Euroimmun	2.2 IU/mL	n.d.	42	99	

^a^ further comparison to other assays not mentioned in this study; genotyping was performed by sequencing analysis described in the individual studies; cohort c: individuals from an unaffected village, no acute illness. rating: +++: most sensitive assay, ++: second most sensitive assay, +: third most sensitive assay.

## Supplementary Materials

The following are available online at www.mdpi.com/1999-4915/8/8/232/s1, Figure S1: Comparison of the linearity and sensitivity of the different anti-HEV IgM assays and the all antibody assay (half-logarithmic scale), Figure S2: Comparison of the linearity and sensitivity of the different anti-HEV IgG assays (half-logarithmic scale).
