# Supplementary material for: Monitoring of Anti-Hepatitis E Virus Antibody Seroconversion in Asymptomatically Infected Blood Donors: Systematic Comparison of Nine Commercial Anti-HEV IgM and IgG Assays
Source: Viruses. 2016 Aug 22;8(8):232. doi: 10.3390/v8080232 (PMC4997594; doi:10.3390/v8080232)
Supplement: Supplementary file 1 [file viruses-08-00232-s001.pdf]

# Supplementary Materials: Monitoring of Anti-Hepatitis E Virus Antibody Seroconversion in Asymptomatically Infected Blood Donors: Systematic Comparison of Nine Commercial Anti-HEV IgM and IgG Assays

**Tanja Vollmer, Juergen Diekmann, Matthias Eberhardt, Cornelius Knabbe and Jens Dreier**

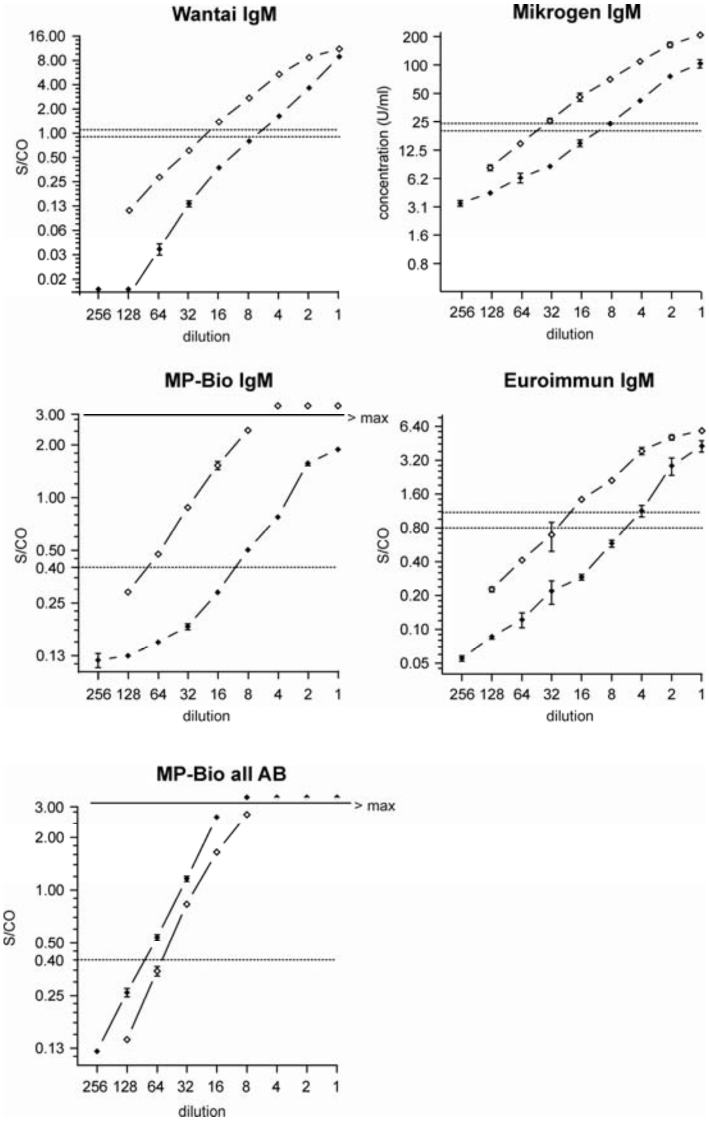


**Figure S1.** Comparison of the linearity and sensitivity of the different anti-HEV IgM assays and the all antibody assay (half-logarithmic scale). Analytical sensitivities for each assay were determined by a twofold dilution series of the WHO-Ref (◆ WHO: dilution 1:1 to 1:256) and the HEV IgM positive sample of donor 6 (◇ DS: dilution 1:1 to 1:128). The dotted horizontal line represents the particular cut-off values for each assay; the solid horizontal line separates test results with S/CO measurements above the linearity range of the assay (> max). All values are given as mean values ± standard deviation (SD).

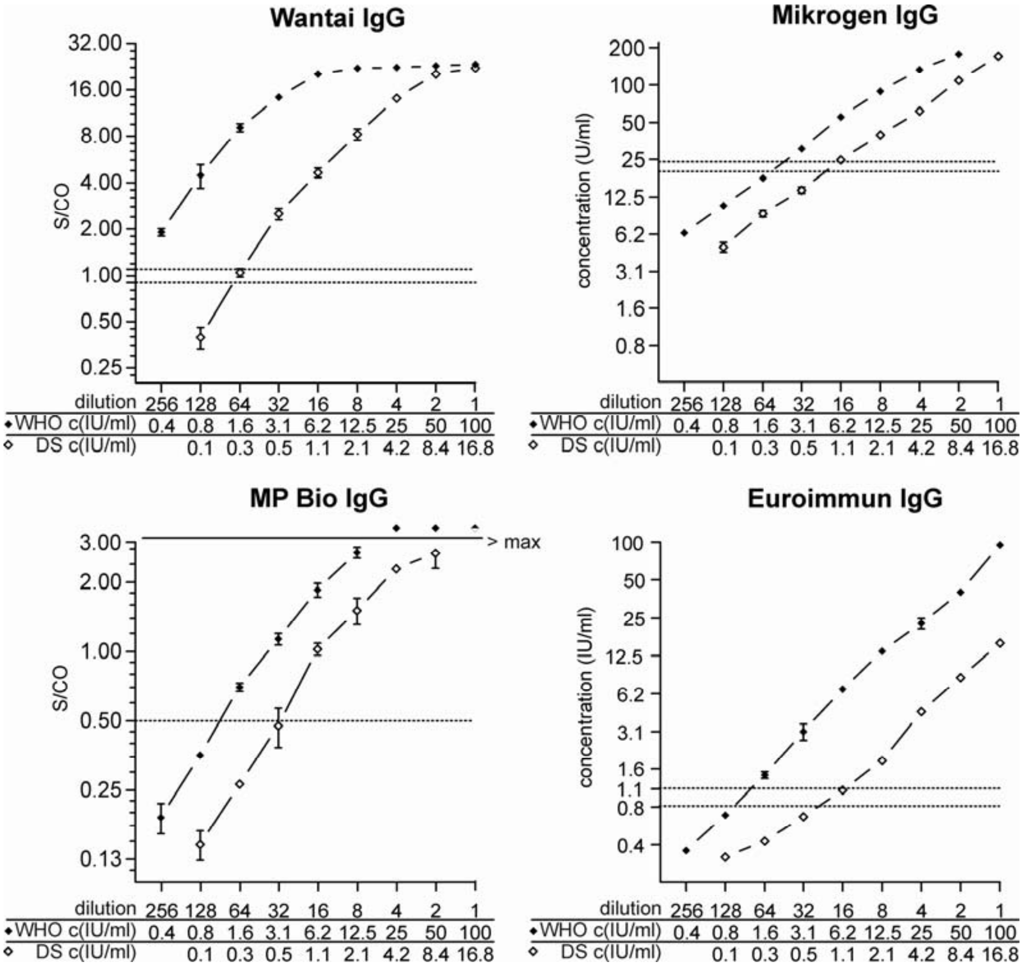


**Figure S2.** Comparison of the linearity and sensitivity of the different anti-HEV IgG assays (half-logarithmic scale). Analytical sensitivities for each assay were determined by a twofold dilution series of the WHO-Ref (◆) WHO: dilution 100 IU/mL–0.4 IU/mL (factor 1:1 to 1:256) and the HEV IgG positive sample of donor 6 (◇ DS: dilution 16.8 IU/mL–0.1 IU/mL (factor 1:1 to 1:128). The dotted horizontal line represents the particular cut-off values for each assay; the solid horizontal line separates test results with S/CO measurements above the linearity range of the assay (> max). All values are given as mean values ± standard deviation (SD).

© 2016 by the author; licensee MDPI, Basel, Switzerland. This article is an open access article distributed under the terms and conditions of the Creative Commons Attribution (CC-BY) license (http://creativecommons.org/licenses/by/4.0/).
